# Cytoplasmic location of NR4A1 in aggressive lymphomas is associated with a favourable cancer specific survival

**DOI:** 10.1038/s41598-018-32972-4

**Published:** 2018-09-28

**Authors:** Karoline Fechter, Julia Feichtinger, Katharina Prochazka, Julia Judith Unterluggauer, Katrin Pansy, Elisabeth Steinbauer, Martin Pichler, Johannes Haybaeck, Andreas Prokesch, Hildegard T. Greinix, Christine Beham-Schmid, Peter Neumeister, Gerhard G. Thallinger, Alexander J. A. Deutsch

**Affiliations:** 1 Division of Hematology, Department of Internal Medicine, Medical University Graz, Graz, Austria; 2 Institute of Computational Biotechnology, Graz University of Technology, Graz, Austria; 3 BioTechMed Omics Center Graz, Graz, Austria; 4 Institute of Pathology, Medical University Graz, Graz, Austria; 5 Division of Oncology, Department of Internal Medicine, Medical University Graz, Graz, Austria; 6 Department of Pathology, Otto von Guericke University Magdeburg, Magdeburg, Germany; 7 Institute of Pathology, Medical University Innsbruck, Innsbruck, Austria; 8 Institute of Cell Biology, Histology and Embryology, Medical University Graz, Graz, Austria

## Abstract

The nuclear orphan receptor *NR4A1* functions as tumour suppressor in aggressive lymphomas by pro-apoptotic genomic and non-genomic effects. Here, we immunohistochemically studied the clinico-pathological relevance of NR4A1 protein expression patterns in a cohort of 60 diffuse large B cell lymphoma (DLBCL) patients and non-neoplastic lymph nodes. We observed a significant association between high cytoplasmic NR4A1 and favourable cancer-specific survival and the germinal centre B cell-like subtype, respectively. Moreover, the percentage of lymphoma cells exhibiting cytoplasmic NR4A1 significantly correlated to those showing cleaved caspase 3. Complementary, functional profiling using gene set enrichment of Reactome pathways based on publicly available microarray data was applied to determine pathways potentially implicated in cytoplasmic localization of NR4A1 and validated by means of semi quantitative real-time PCR. The pathway analysis revealed changes in the ERK1/2 pathway, and this was corroborated by the finding that high cytoplasmic NR4A1 was associated with higher expression of ERK1/2 targets in our cohort. These data indicate that high cytoplasmic NR4A1 is associated with a favourable lymphoma-specific survival and highlights the importance of NR4A1 expression patterns as potential prognostic marker for risk assessment in aggressive lymphomas.

## Introduction

Diffuse large B cell lymphomas are the most common subtype of aggressive B cell lymphomas, accounting for 30–40% of all cases in adults. DLBCL arise either *de novo* as primary DLBCL or by histologic transformation of less aggressive B-cell non-Hodgkin lymphoma (NHL) -subtypes, i.e. follicular lymphoma (FL) as transformed DLBCL^1,2^. Clinical outcome varies depending on different clinical and pathological risk factors with an overall 5-year survival rate of around 50%. Despite improvements in therapy, approximately one third of patients with advanced-stage DLBCL is still unresponsive to therapy or will relapse. Gene expression profiling showed that DLBCLs cluster into three different subtypes based on an expression pattern similar to the cellular origin: germinal centre B cell-like (GCB-DLBCL), activated B cell-like/ or non-germinal centre B cell-like DLBCL (ABC-DBLCL or NGCB-DLBCL) and primary mediastinal B cell lymphoma (PMBL)^3,4^. These subtypes of DLBCLs are associated with distinctly different overall survival rates after immunochemotherapy such as R-CHOP (Rituximab, cyclophosphamide, doxorubicin, vincristine and prednisolone): overall survival is favourable in patients with GCB subtype and PMBL, and inferior in those with the NGCB subtype^3^.

*NR4A1 (Nuclear Receptor Subfamily 4 Group A Member 1, Nur77)*, together with *NR4A2 (Nurr1)* and *NR4A3 (NOR-1)*, belongs to the Nur77 family of nuclear orphan receptors^5^. All three family members are widely expressed in numerous tissues, such as skeletal muscle, adipose tissue, heart, kidney, T cells, liver, and brain. Their activation is generally short lived. The cellular outcome is a stimulus- and cell context-dependent differential activation of NR4A target genes that regulate cell cycle, apoptosis, inflammation, atherogenesis, metabolism, or DNA repair, and, as described more recently, are involved in tumorigenesis. *NR4A1* plays a role in the negative selection of T -lymphocytes, as well as in viral-induced B cell apoptosis^6,7^. In mice, *Nr4a1* was recently identified to function together with *Nr4a3* as tumour suppressor in acute myeloid leukaemia (AML)^8^. Moreover, we previously demonstrated a significant reduction of both - *NR4A1* and *NR4A3 –* in major B cell neoplasms like chronic lymphocytic leukaemia (CLL), FL and DLBCL compared to normal controls^9^. Survival analysis revealed that low *NR4A1* expression is associated with poor cancer-specific survival in DLBCL^9^, which could be confirmed in an independent cohort of the publicly available gene expression dataset of Lenz *et al*.^10^. Overexpression of *NR4A1* induced apoptosis in several lymphoma cell lines, and these pro-apoptotic effects were mediated by the nuclear properties of NR4A1 as transcription factor^9,11^.

It has been reported that the pro-apoptotic function of NR4A1 can also be facilitated by its translocation from the nucleus to the cytoplasm thereby causing mitochondrial apoptosis^12,13^. This cytoplasmic localization is regulated by posttranslational phosphorylation within different positions of the amino acid sequence of NR4A1^7^. Based on these findings, we aimed to examine the clinico-pathological relevance of cytoplasmic expression patterns of NR4A1 in DLBCLs. Here, we demonstrate for the first time that a varying percentage of DLBCL cells showed NR4A1 protein expression in the cytoplasm, whereas none of the non-neoplastic germinal centre B cells expressed cytoplasmic NR4A1. Importantly, high cytoplasmic NR4A1 levels were associated with favourable lymphoma-specific survival and with a higher amount of lymphoma cells exhibiting cleaved caspase 3. Moreover, we found significantly increased expression of genes regulated by extracellular signal-related kinase 1/2 (ERK1/2) in DLBCLs exhibiting high cytoplasmic NR4A1 content, indicating that this pathway could cause or at least contribute to the translocation and subsequent induction of apoptosis by NR4A1.

## Results

### Higher cytoplasmic NR4A1 correlates with the GCB-DLBCL subtype and increased survival

**Figure 1:**
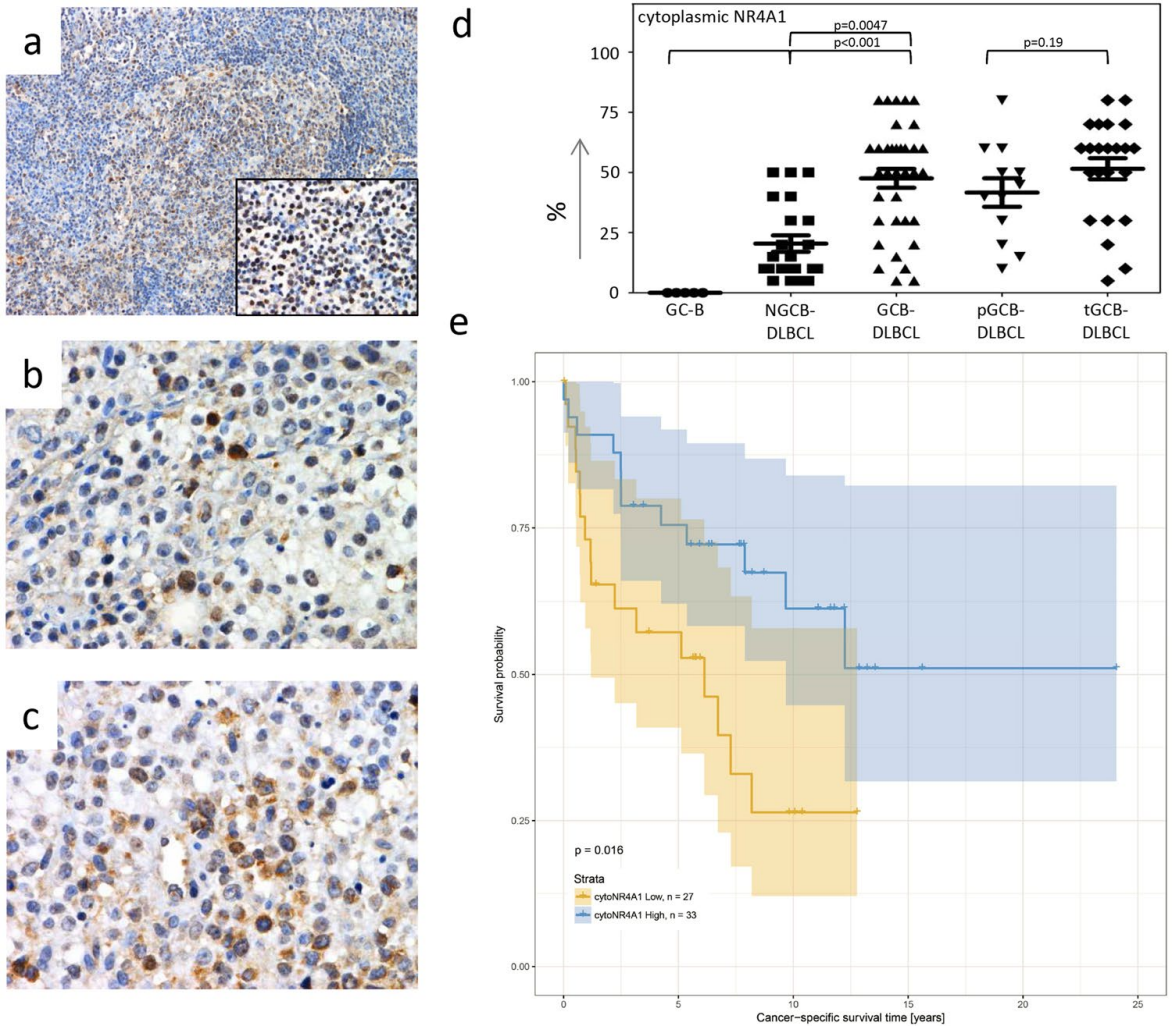
Immunohistochemical NR4A1 analysis and overall survival. (**a**–**c**) NR4A1 expression in (**a**) non-neoplastic lymph nodes (magnification 100x for the overview and magnification 400x for the big picture). (**b**) NGCB-DLCBL (magnification 400x) and (**c**) GCB-DLBCL (magnification 400x) determined by immunohistochemical analysis. All images were captured by using an Olympus BX51 microscope and an Olympus E-330 camera. (**d**) Scatter plot of the distribution of the cytoplasmic levels of NR4A1 in GC-B* (n = 5), NGCB- (n = 22) and GCB-DLBCL (n = 35) consisting of GCB-DLBCL (pGCB-DLBCL, n = 12) and transformed GCB-DLBCL (tGCB-DLBCL, n = 23) as two different subtypes of GCB-DLBCL. (**e**) Kaplan Meyer graphical illustration of the cancer-specific survival classified by levels of cytoplasmic NR4A1 of all lymphoma patients. Patients with high cytoplasmic NR4A1 expression are shown in blue and with low cytoplasmic expression in yellow. *GC-B denotes germinal centre B-cells, which were isolated from non-neoplastic tonsils.

We performed a comprehensive histology-based study on cytoplasmic NR4A1 staining pattern in our cohort of DLBCL (n = 60). The patients’ characteristics are summarized in Supplementary Table 1. As we have previously reported, total (cytoplasmic and nuclear) NR4A1 expression is significantly reduced in aggressive lymphoma specimens when compared to non-neoplastic germinal centre B cells (GC-B)^9^. However, while we only detected nuclear and no cytoplasmic NR4A1 in the non-neoplastic GC-B, the percentage of aggressive lymphoma cells with NR4A1 in the cytoplasm varied between 5–80% (on average 27.1% for NGCB- and 48.5% for GCB-DLBCL, p = 0.0004, Fig. 1a–d). By comparing cytoplasmic to nuclear NR4A1, we did not detect any correlation between the two different expression patterns (Spearman’s rho = 0.204, p = 0.112, Supplementary Figure 1). By further analysis of the two different subtypes of GCB-DLCBL, we detected a similar cytoplasmic NR4A1 extent in primary (*de novo*) GCB-DLCBL (pGCB-DLBCL) as compared to the transformed subtype (tGCB-DLBCL) (on average 41.7% vs. 52.4%, p = 0.19, Fig. 1d). By stratifying the patient cohort into two groups using the median percentage of lymphoma cells (median = 40%) exhibiting a cytoplasmic NR4A1 staining (as opposed to nuclear or no NR4A1), a significant association between high cytoplasmic NR4A1 level and favourable cancer-specific survival was observed (n = 60, p = 0.016, log-rank test, Fig. 1e). Excluding the tGCB-DLCBL cases, we obtained a similar result (n = 37, p = 0.036, log-rank test, Supplementary Figure 2). High cytoplasmic NR4A1 occurred more frequently in GCB-DLBCL compared to the NGCB-subtype (71.4% vs. 31.8%, p = 0.0057, Fisher’s exact test, Table 1). In addition, the cancer-specific survival analysis based on the cytoplasmic NR4A1 expression pattern (p = 0.016, Fig. 1e) showed a slightly lower p-value than that of the same patients based on *NR4A1* expression (p = 0.032, Supplementary Figure 3a), which we described in our previous work^9^. Notably, omitting the tGCB-DLCBL cases led here to loss of significance when basing the survival analysis on *NR4A1* expression (n = 37, p = 0.108, log-rank test, Supplementary Figure 3b). Here, it should be noted that in 30% (18/60) of DLBCL- and 35.1% (13/37) of *de novo* DLBCL-cases the *NR4A1* gene expression status (high vs. low) does not coincide with the cytoplasmic NR4A1 expression pattern (high vs. low). Additionally, the comparison of cytoplasmic NR4A1 staining pattern to RNA expression levels showed no correlation (Spearman’s rho = 0.114, p = 0.471, Supplementary Figure 4a), whereas the nuclear content significantly correlated to RNA expression levels (Spearman’s rho = 0.650, p < 0.001, Supplementary Figure 4b). Taking altogether, these data indicate that high cytoplasmic NR4A1 levels might not only be caused by high *NR4A1* expression. Multivariate analysis (n = 57, three omitted as subtype not available, Table 2) revealed subtype and age as independent prognostic factors to be significantly associated with survival (p < 0.05). In addition, cytoplasmic NR4A1 levels and tumor stage were found to be borderline significant. Advanced tumour stage (3 or 4), the NGCB subtype, increasing age, and low cytoplasmic NR4A1 levels present poor prognostic factors.

**Table 1:** Cytoplasmic NR4A1 expression in DLBCL.

	Low (<40%)	High (>40%)	p-value
NGCB-DLBCL	15	7	0.0057
GCB-DLBCL	10	25	

The frequency of cytoplasmic NR4A1 expression was stratified by the median percentage of lymphoma cells exhibiting cytoplasmic NR4A1 expression (40%). Statistical calculation was performed using the Fisher’s exact test.

**Table 2:** Cox Proportional Hazards Regression Analysis.

	1^st^ group	2^nd^ group	Univariate				
			HR	Lower 95% CI	Upper 95% CI	P-value	
Age	continuous		1.070	1.031	1.110	**0.0004**	***
Sex	male, n = 32	female, n = 25	0.641	0.296	1.388	0.2590	
Stage	1–2, n = 22	3–4, n = 35	1.520	0.682	3.388	0.3060	
cyto NR4A1	low, n = 25	high, n = 32	0.385	0.175	0.847	**0.0176**	*
Subtype	GCB, n = 35	NGCB, n = 22	9.556	3.743	24.390	<**0.0001**	***
			**Multivariate**				
Age	continuous		1.066	1.011	1.123	**0.0173**	*
Sex	male, n = 32	female, n = 25	0.533	0.189	1.503	0.2342	
Stage	1–2, n = 22	3–4, n = 35	2.735	1.006	7.439	**0.0487**	(*)
cyto NR4A1	low, n = 25	high, n = 32	0.378	0.148	0.966	**0.0422**	(*)
Subtype	GCB, n = 35	NGCB, n = 22	4.790	1.538	14.922	**0.0069**	**

Univariate and multivariate Cox proportional hazard ratio calculation. Hazard ratios (HR) are stated for the 2^nd^ group relative to the 1^st^ group. (*)borderline significant.

*p-value ≤ 0.05.

**p-value ≤ 0.01.

***p-value ≤ 0.001.

### Cytoplasmic NR4A1 is associated with higher occurrence of apoptotic lymphoma cells

**Figure 2:**
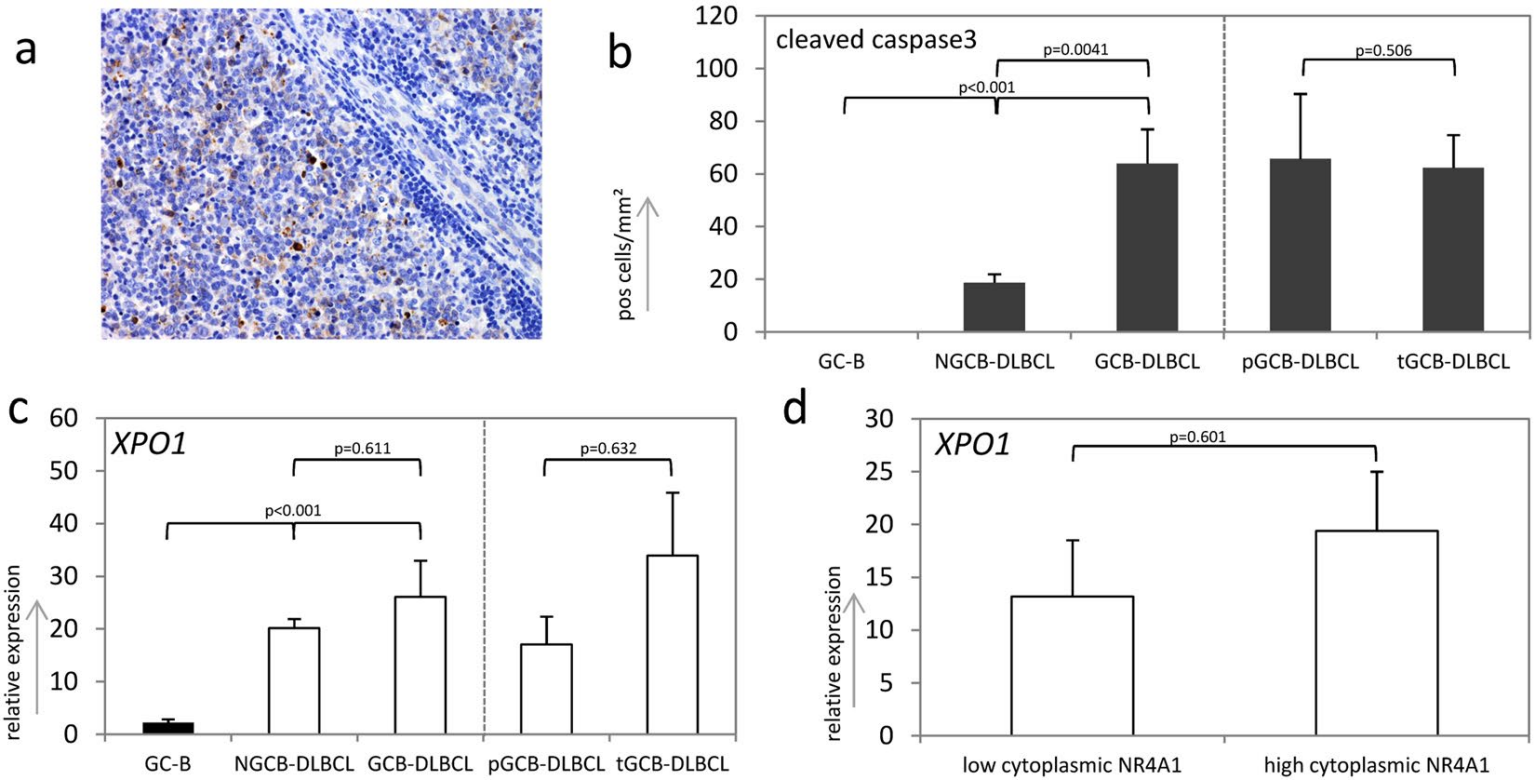
Immunohistochemical analysis of cleaved caspase 3 and expression levels of XPO 1: (**a**) Immunohistochemical stain for cleaved caspase 3 in a representative GCB-DLBCL sample. (**b**) Number of cells exhibiting a cleaved caspase 3 depicted in cells/mm^2^ in non-neoplastic GC-B* (n = 4), NGCB-DLBCL (n = 19) and GCB-DLBCL (n = 33) samples and samples of the two different GCB subtype namely primary GCB- (pGCB-DLBCL, n = 12) and transformed GCB-DLBCL (tGCB-DLBCL, n = 21). Each bar represents the mean values of each group ± standard error of the mean (SEM). (**c**) Relative *XPO1* expression levels in non-neoplastic lymph nodes (GC-B) (n = 4), NGCB-DLBCL (n = 19), GCB-DLBCL (n = 33) and the two different GCB subtype namely primary GCB- (pGCB-DLBCL, n = 12) and transformed GCB-DLBCL (tGCB-DLBCL, n = 21) as determined by RQ-PCR. Each bar represents the mean values of expression levels ± standard error of the mean (SEM) (**d**) Relative *XPO1* expression levels as determined by RQ-PCR in set of low (n = 26) and high (n = 26) cytoplasmic NR4A1 expressing DLCBL specimens. *GC-B denotes germinal centre B-cells, which were isolated from non-neoplastic tonsils.

Several reports underpin the role of cytoplasmic NR4A1 in the induction of mitochondrial apoptosis^12,13^. In order to gain insight into the influence of cytoplasmic NR4A1 on cell death, we performed an immunohistochemistry-based analysis for cleaved caspase 3 in our cohort of aggressive lymphoma patients. The percentage of cytoplasmic NR4A1 expressing cells significantly correlated with the number of lymphoma cells exhibiting cleaved caspase 3 (Spearman’s rho = 0.741, p < 0.001). This data suggests that cytoplasmic NR4A1 might induce apoptosis in aggressive lymphomas. In addition, we could detect cleaved caspase 3 in both, NGCB- and GCB-DLBCL subtypes (Fig. 2a). Importantly, comparison of the number of positive cells/mm^2^ present in tissue specimens of NGCB- and GCB-DLBLC, respectively, revealed a clear association with the GCB-DLBCL subtype: 3.3-fold more cleaved caspase 3 could be detected in these samples (p = 0.0041, Fig. 2b), whereas non-neoplastic GC-B did not exhibit any cleaved caspase 3 (p < 0.001, Fig. 2b). Once more, by comparing primary to transformed GCB-DLCBL we detected no statistically significant difference in the average number of cells exhibiting cleaved caspase 3 in both subtypes (pGCB- and tGCB-DLBCL, p = 0.506, Fig. 2b).

It is known that Exportin 1 (XPO1) is crucial for translocating NR4A1 from the nucleus to the cytoplasm^14^. To clarify whether high cytoplasmic NR4A1 levels correspond to *XPO1* overexpression, we determined the relative *XPO1* expression and set it in relation to the occurrence of cytoplasmic NR4A1 in our cohort of aggressive lymphomas. Compared to GC-B, GCB- and NGCB-DLBCL exhibited a significantly higher *XPO1* expression (22.4 fold, p < 0.001 for GCB-DLBCL, 19.8 fold p < 0.001 for NGCB-DLBCL, Fig. 2c). *XPO1* was not statistically differentially expressed in pGCB-DLCBL compared to tGCB-DLCBL (p = 0.632, Fig. 2c). Furthermore, no statistically significant differences in *XPO1* expression were detected by comparing lymphomas with high cytoplasmic to specimens with low or no cytoplasmic NR4A1 levels (p = 0.601, Fig. 2d) suggesting that overexpression of *XPO1* might not be the sole reason for the cytoplasmic localization of NR4A1 in aggressive lymphomas.

### High cytoplasmic NR4A1 is associated with increased expression of ERK1/2 target genes in aggressive lymphomas

Based on the association of cytoplasmic NR4A1 expression levels with GCB-DLBCL (71.4% in GCB vs. 31.8% in NGCB, p = 0.0057, Fisher’s exact test, Table 1), we determined differentially expressed genes by comparing GCB- and NGCB-DLCBL in the publicly available dataset of Lenz *et al*.^10^, containing samples from 200 R-CHOP-treated patients. 959 genes (1542 features) were found to be differentially expressed based on an adjusted p-value < = 0.05 and abs(log_2_FC) >log_2_(1.5) (Supplementary Table 2, 384 upregulated and 575 downregulated). These include among others *BCL6*, *BCL7A*, *CCND2*, *IRF4*, *CD44*, *CFLAR*, *IL16*, *LMO2*, and *MYBL1*, which have been reported previously as differentially expressed between the subtypes^15,16^.

We further screened for possible mechanisms causing NR4A1 translocation to the cytoplasm in this subtype by functional profiling. Therefore, we performed two complementary functional profiling approaches: a gene ontology analysis using the 959 differentially expressed genes and a gene set enrichment analysis of Reactome pathways. With the first approach we performed an enrichment analysis to identify GO terms that are over-represented based on annotations for the differentially expressed genes, whereas the latter enabled us to detect pathways (gene sets) exhibiting statistically significant, concordant changes between the two DLBCL subtypes. As gene set enrichment analyses take all genes into account, itcan provide higher sensitivity in detecting also moderate changes.

The GO analysis resulted in 308 significantly enriched GO terms (p-value < = 0.01, Supplementary Table 3). Many terms were associated with immune response, in particular with B cells, arising from the difference in cellular origin of the two DLCBL subtypes (GCB vs. NGCB). 11 GO terms were associated with apoptosis, suggesting this process to be different in the two subtypes, which is in agreement with our caspase 3 results. As 15 GO terms were associated with kinase activity and/or protein phosphorylation, we searched for more specific GO terms associated with mechanisms that possibly lead to NR4A1 translocation. It is known that extracellular signal-related kinase 1/2, protein kinase B (PKB, AKT), c-Jun N-terminal kinase (JNK), and ribosomal S6 kinase (RSK) can post-translationally phosphorylate NR4A1, resulting in cytoplasmic localization of NR4A1^6,7^. Indeed, 4 GO terms were associated with the ERK1/2 or the MAPK (mitogen-activated protein kinase) cascade and 2 GO terms with AKT signalling, suggesting differential activation of these processes in the two subtypes.

Pathway analysis yielded 110 significantly enriched Reactome pathways (q-value < = 0.05, Supplementary Table 4). Here, the pathways ‘phosphatidylinositide 3 kinase (PI3K)/AKT activation’, ‘phosphatidylinositol (3,4,5) triphosphate (PI3P) activates AKT signalling’, ‘PI3K/AKT Signalling in Cancer’, and ‘Constitutive Signalling by Aberrant PI3K in Cancer’ were found to be significantly enriched. To assess how genes associated with the enriched Reactome pathways relate to each other, we generated a gene/pathway matrix (Supplementary Table 5) and used clustering to identify functional related gene groups (Supplementary Figure 5). Many related pathways, such as pathways associated with receptors that signal through AKT, were also determined to be significantly enriched, as can be seen in the highlighted cluster of Supplementary Figure 5.

**Figure 3:**
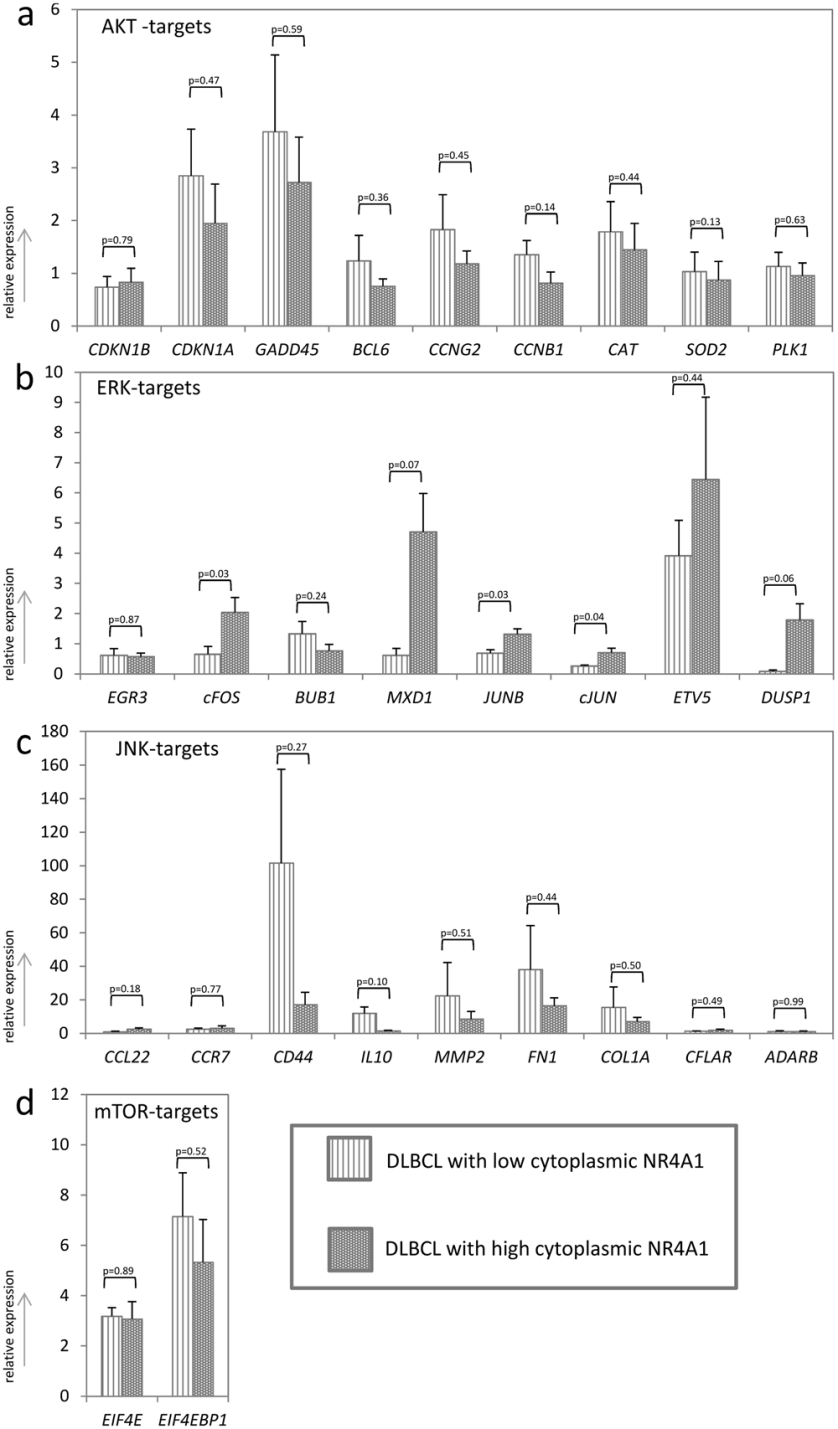
Expression analysis of AKT-, ERK1/2-, JNK- and mTOR- target genes in low (n = 9) and high (n = 8) cytoplasmic expressing aggressive lymphoma specimens determined by RQ-PCR. (**a**) *CDKN1B, CDKN1A, GADD45, BCL6, CCNG2, CCNB1, CAT* *SOD2* and *PLK1* as AKT target genes (based on literature). (**b**) *EGR3, cFOS, BUB1, MXD1, JUNB, cJUN, ETV5* and *DUSP1* as ERK1/2 target genes (based on literature). (**c**) *CCL22*, *CCR7*, *CD44*. *IL10, MMP2*, *FN1*, *COL1A*, *CFLAR* and *ADARB* as JNK target genes (based on literature). (**d**) *EIF4E* and *EIF4EBP1* as mTOR target genes (based on literature). Each bar represents the mean values of expression levels ± standard error of the mean (SEM).

**Figure 4:**
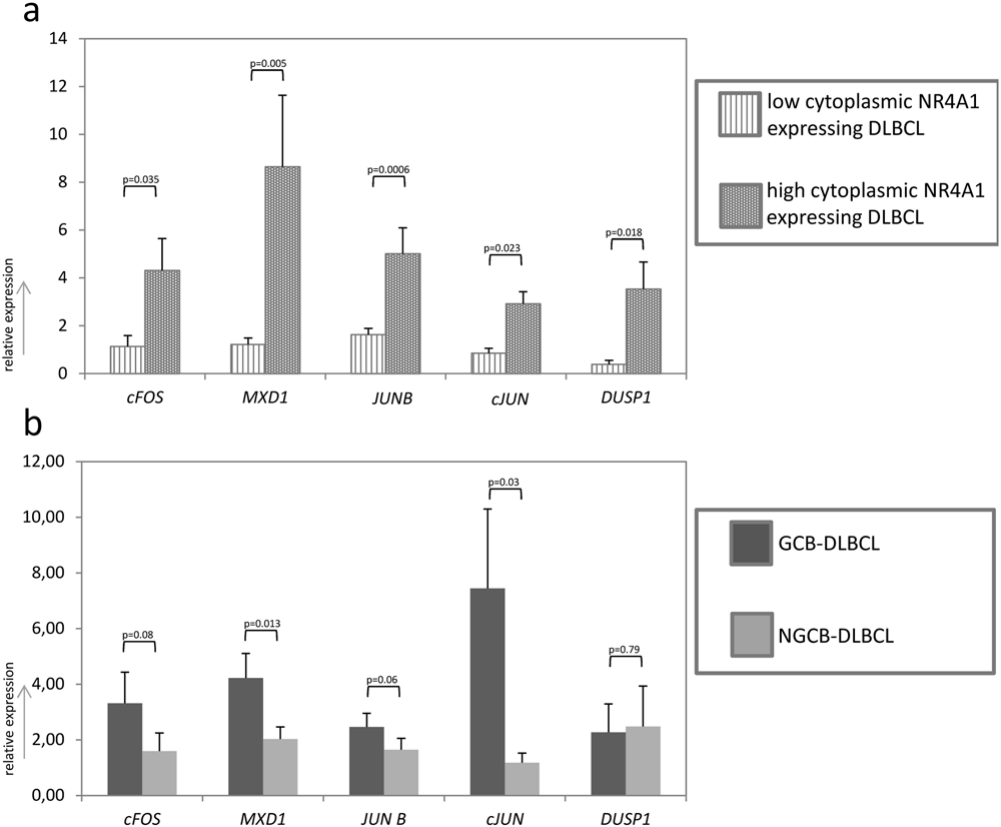
Expression analysis of ERK1/2 target genes. (**a**) Relative expression levels of *cFOS*, *MXD*, *JUN B*, *cJUN* and *DUSP1* in low (n = 21) and high (n = 30) cytoplasmic expressing DLBCL determined by RQ-PCR. (**b**) Relative expression levels of *cFOS*, *MXD*, *JUNB*, *cJUN* and *DUSP1* in GCB- (n = 29) and NGCB- (n = 22) DLBCL determined by RQ-PCR. Each bar represents the mean values of expression levels ± standard error of the mean (SEM).

To assess, whether these pathways are indeed changed in cytoplasmic NR4A1 high compared to cytoplasmic NR4A1 low DLCBL, we independently chose several target genes known to be representative for the respective pathways identified by GO and pathway analyses as well as for pathways know to be implicated in lymphoma development. An explorative gene expression analysis for *EGR3, cFOS, BUB1, MXD1, JUNB, cJUN, ETV5* and *DUSP1* - genes known to be regulated by ERK1/2^17^ - as well as for - *CDKN1B, CDKN1A, GADD45, BCL6, CCNG2, CCNB1, CAT, SOD2* and *PLK1* - genes known to be regulated by AKT^18–20^ was performed in our lymphoma cohort. Analyses were set in relation to the staining pattern of NR4A1 and DLBCL subtype on selected cases with either extremely low cytoplasmic NR4A1 (below 20%, n = 9 consisting of 6 NGCB- and 3 GCB-DLBCL) or high cytoplasmic NR4A1 (above 80%, n = 8 consisting of 2 NGCB- and 6 GCB-DLBCL). Additionally, we analysed two other important pathways involved in lymphomagenesis, namely JNK^21,22^ and mammalian target of rapamycin (mTOR)^23–25^ by gene expression profiling: *CCL22, CCR7, CD44, IL10, MMP2, FN1, COL1A, CFLAR* and *ADARB* for JNK (based on the Ingenuity Pathway Analysis tool) and *EIF4E* and *EIF4EBP1* for mTOR^26^. Remarkably, five of the eight ERK target genes investigated showed increased expression in cytoplasmic high NR4A1 DLBCL: Expression of *cFos*, *JUNB*, *cJUN*, *MXD1*, and *DUPS1* was 5.1 fold, 1.9 fold, 2.6 fold (p < 0.041), 7.6 fold, and 21.1 fold higher (p < 0.071, Fig. 3b), respectively. None of the other genes investigated were differentially expressed in these specimens (Fig. 3a, c-d). In order to rule out that the higher expression of *cFOS*, *MXD1*, *JUNB*, *cJUN*, and *DUSP1* in cytoplasmic NR4A1 expressing DLBCL is due the stringent conditions applied (below 20% and above 80%), we increased the number of lymphoma cases in our patient cohort (n = 34 consisting of 12 low and 22 high cytoplasmic NR4A1 expressing DLBCL - so a total of 51 DLBCL cases) by classifying all specimens as having lower or higher cytoplasmic NR4A1 expression than the median of 40% of lymphoma cells positive for NR4A1. Here again, expression of *cFOS, MXD1*, *JUNB, cJUN*, and *DUSP1* was significantly higher (3.7 fold, 7.1 fold, 3.1 fold, 3.4 fold and 9.1 fold, respectively) in lymphomas with high cytoplasmic NR4A1 content (p < 0.036, Fig. 4a). Additionally, expression of *cFOS, MXD1, JUNB*, and *cJUN* was 2.1, 2.1, 1.5, and 6.3 fold higher, respectively, in GCB-DLCBL compared to NGCB-DLBCL (*MXD1* and *cJUN p* < *0.03*, tendency in *cFOS* and *JUNB* p < 0.081, Fig. 4b). Importantly, by comparing pGCB-DLBCL to tGCB-DLCBL none of the investigated genes was found to be statistically significant differentially expressed (p > 0.17, Supplementary Figure 6). These data suggest that the ERK1/2 pathway activation might be implicated in NR4A1 nuclear export in DLBCLs, which has already been shown in lung cancer cells as well as human primary T- and kidney cells^27–29^.

## Discussion

This study was designed to investigate the subcellular expression pattern of NR4A1 in aggressive lymphomas. There is growing evidence that NR4A1 plays an important role in human tumorigenesis and lymphomagenesis^6,7,9,11^ and that the apoptotic effects of a number of anti-tumoral agents are mediated by the cytoplasmic function of NR4A1^6,7^. Furthermore, NR4A1 is rapidly transcriptionally induced in response to B cell receptor signalling and is expressed in murine germinal centre B cells^30,31^. Even though this confirms our observation, data on the clinico-pathological relevance of intracellular localization (cytoplasmic and nuclear) of NR4A1 in tumours and especially in lymphomas are scarce. To the best of our knowledge, only one study has dealt with protein levels in cytoplasm of another member of the Nur77 family of nuclear orphan receptors, namely NR4A2. In that study, high levels of cytoplasmic NR4A2 were associated with adverse outcome in bladder cancer patients^32^. Contrary to that, our survival analysis clearly demonstrated that high cytoplasmic NR4A1 levels were associated with a favourable clinical course in DLBCLs by performing uni- and multivariate survival analyses. Survival analysis using cytoplasmic NR4A1 levels exhibited a lower p-value than using *NR4A1* expression levels, suggesting cytoplasmic NR4A1 as a better surrogate marker in aggressive lymphomas. Nevertheless, it is noteworthy that cytoplasmic NR4A1 is highly associated with distinct GCB-DLBCL subtypes. Moreover, cytoplasmic NR4A1 is - when compared to the univariate analysis - only borderline significant in the multivariate analysis, which itself is limited by the relatively small cohort size. With respect to primary DLBCL samples, it is possible that some specimens characterized as such, represent rather transformed specimens where the patients did not underwent proper physical examination prior to diagnosis of DLBCL. Therefore, a more detailed investigation is needed to show whether high cytoplasmic NR4A1 is indeed an independent factor for disease outcome or a characteristic of the GCB subtype.

Together with the fact that rituximab and doxorubicin - two components of the R-CHOP regimen - induce *NR4A1* expression and that NR4A1 is mandatory for viral and BCR (B cell receptor) mediated B cell apoptosis^33–37^, it could be speculated that NR4A1, especially cytoplasmic NR4A1, influences the therapeutic effect of the cytostatic therapeutic strategies. We also observed that high cytoplasmic NR4A1 levels occurred with a higher incidence in GCB-DLBCL than in NGCB-DLBCL. Several studies have demonstrated that NR4A1 targets the mitochondria in the cytoplasm, where it binds the hydrophobic groove of the BCL-2 protein, leading to a change in the BCL-2 conformation that changes its function from a cytoprotective to a cytodestructive molecule triggering cytochrome C release and apoptosis^12,13^. In our lymphoma cohort, we showed that the level of cytoplasmic NR4A1 was associated with the number of lymphoma cells exhibiting a cleaved caspase 3, suggesting that NR4A1 might target the mitochondria in aggressive lymphomas and thereby induces apoptosis. In addition, we observed that the number of lymphoma cells expressing cleaved caspase 3 was significantly higher in GCB-DLBCL. Two other comprehensive studies also demonstrated cleavage of caspase 3 in nodal DLBCL, but they did not show any association with the GCB subtype^38,39^. GO and pathway analysis also determined an enrichment of genes involved in apoptosis. This aligns with our results of the cleaved caspase 3 assay and provides further support for the hypothesis that cytoplasmic NR4A1 is possibly an important factor in the survival of aggressive lymphoma patients.

We determined that *XPO1* – encoding a protein known to transport NR4A1 from nucleus to cytoplasm^14^ – was overexpressed in the GCB-DLBCL and NGCB-DLBCL subtype compared to GC-B control cells. Overexpression of *XPO1* was shown in primary mediastinal B cell lymphomas and classic Hodgkin lymphomas, mantle cell lymphomas^40,41^, as well as in indolent lymphomas and myeloid neoplasms^42,43^. However, to the best of our knowledge, *XPO1* overexpression has not been described in DLBCL so far. A significant number of NGCB- and GCB-DBCL specimens have been reported to exhibit a copy number gain in 2p15, the chromosomal locus of *XPO1*^44^, suggesting this as a mechanism for *XPO1* overexpression in DLBCL. XPO1 is a new target in cancer therapy based on the development of several XPO1 inhibitors only targeting tumour cells but not normal hematopoietic cells. Inhibitors like KPT-251 and KPT-330 (Selinexor) displayed anti-proliferative and pro-apoptotic activities in various haematological malignancies in both *in vivo* and *in vitro* settings^45–47^. Importantly, Selinexor, an orally available irreversible XPO1 inhibitor, showed promising results with a response rate of 31% in patients with advanced NHL participating in a clinical phase I trial^48^. However, a correlation between *XPO1* overexpression and levels of cytoplasmic NR4A1 could not be observed, suggesting that other factors/mechanisms might trigger NR4A1 translocation to the cytoplasm.

To identify other mechanisms that could lead to NR4A1 translocation to the cytoplasm and therefore to elevated apoptosis, we performed functional profiling of GCB- and NGCB-DLBCL microarray expression data, as the GCB subtype is highly associated with high cytoplasmic NR4A1 levels. GO and pathway analysis revealed an enrichment of changes in ERK1/2 and AKT signalling. A number of kinases such as JNK, AKT, RSK, and ERK1/2^27–29,49–52^ have already been shown to phosphorylate NR4A1, thereby causing translocation to the cytoplasm. Our explorative gene expression analysis of ERK1/2-, AKT-, JNK-, and mTOR-target genes demonstrated that five of eight ERK1/2 target genes investigated showed higher expression in DLBCLs with high cytoplasmic NR4A1 levels. The obtained results point to an association between activated ERK1/2 and cytoplasmic localization of NR4A1 and indicate that ERK1/2 signalling may play a role in cytoplasmic localization of NR4A1 in aggressive lymphomas.

This study provides the first evidence that the subcellular localization of NR4A1 can be clearly and robustly correlated to different lymphoma subtypes and cancer-specific survival of aggressive lymphoma patients. Likewise, the cytoplasmic NR4A1 expression patterns represent a potentially useful marker for risk stratification and therapeutic intervention.

## Materials and Methods

### Patient samples

Tumour specimens consisted of lymph node samples of 60 patients with DLBCLs, including 37 de novo and 23 transformed lymphoma samples, receiving a Rituximab containing regimen at the Division of Hematology, Medical University of Graz between 2000 and 2010. Examined transformed DLBCL samples with an underlying diagnosis of follicular lymphoma only contained high grade component. Clonal relationship between an initial FL sample and the resulting transformed DLBCL sample was determined by immunoglobulin heavy chain rearrangement PCR comparing the respective paired specimens. Sample cohort, obtained from the Institute of Pathology, Medical University of Graz, was the same as already published^9^. For this study, samples were re-evaluated, and the median follow-up time was increased until May 2016 (median 5.84 years). All samples represented DLBCLs using the WHO classification^53^. Based on the IHC profiles according to the Hans algorithms^54^, all cases were classified as follows: 12 cases were categorized as germinal centre subtype, 22 as non-germinal-centre B-cell like, and three as non-classifiable. In these three cases, re-classification was not possible because of lacking tumour material. As transformed DLBCL samples originating from FLs exhibited a similar expression pattern as GCB-DLCBL samples^55^, 23 transformed DLBCL samples were added to this subtype (Supplementary Table 1). For this retrospective study, we used patient specimens obtained for routine diagnostic procedures. Hence, no written informed consent of patients was obtained. This consent procedure was approved by the Ethical Committee of the Medical University Graz (No. 28–517 ex 15/16). All experiments were performed in accordance with relevant guidelines and regulations.

### Survival analysis

To test whether different levels of NR4A1 in the cytoplasm influence clinical outcome, we analysed the association between histological NR4A1 abundance and clinical data of histologically-confirmed aggressive lymphoma patients (summarized in Supplementary Table 1). Patients were categorized into two groups using as a cut-off the median percentage of lymphoma cells exhibiting NR4A1 in the cytoplasm (in the following termed low and high cytoplasmic NR4A1 expression). Survival analysis was performed in R 3.3.3^56^ using the R package ‘survival’^57^. Patients’ cancer-specific survival was calculated with the Kaplan-Meier method, compared by the log rank test. Survival curves were visualized using the R package ‘survminer’^58^. P-values < = 0.05 were considered as statistically significant. The Cox proportional-hazards model was applied to investigate the association between the survival time and clinico-pathological variables (cytoplasmic NR4A1 level, age, sex and stage – univariate and multivariate analysis), thereby determining Hazard ratios (HRs), corresponding 95% confidence intervals (CIs) and p-values. The validity of the Cox proportional-hazards model (assessing the assumption of proportional hazards as well as testing for non-linearity) was checked using the R package ‘survival’^57^, survminer’^58^ and ‘Greg’^59^. P-values < = 0.05 were considered as statistically significant.

### Immunohistochemistry

Formalin-fixed paraffin embedded tissue was stained after pre-treatment with Target Retrieval Solution (Dako, Glostrup, Denmark) using the staining kit K5001 (Dako, Glostrup, Denmark) and the automated stainer intelliPATH FLX® (Biocare Medical, Pacheco, CA, USA). Primary antibody to NR4A1 (clone E-6, 1:200, order number: sc-166166) was purchased from Santa Cruz (Dallas, TX, USA) and primary antibody to cleaved caspase 3 (clone 5A1E, 1:50, order number: 9664 L) from Cell Signaling (Leiden, The Netherlands). To determine the IHC profiles according to the Hans algorithms, primary antibodies to CD10 (clone 56C6, 1:6, Novocastra, Leica Biosystems, Wetzlar, Germany, order number: NCL-CD10–270), MUM1 (clone MUM1p, 1:50, Dako, Glostrup, Denmark, order number: M7259) and BCL-6 (clone GL191E/A8, 1:100, Cell-Marque, Newcastle Upon Tyne, United Kingdom, order number: 227M-96) were applied. For control purposes, tissues known to contain the respective antigens were included (positive controls). Replacement of the primary antibody by normal serum always revealed negative results (negative controls). Immunohistochemical analysis was independently performed by two pathologists according to the following procedure: For determination of the cytoplasmic NR4A1 content and cleaved caspase 3 the whole section was screened for an equal distribution of positive cells. The determination of the percentage of cytoplasmic NR4A1 was done by calculating the average percentage of cytoplasmic NR4A1 positive cells in at least ten high-power-fields (HPFs) (0.242mm^2^ each, field diameter: 555.1 µm). Percentages were rounded to 10%. For the evaluation of the cell number exhibiting cleaved caspase 3, the average of positive cells counted in ten HPF was used to calculate the number of cells in four HPF corresponding to 1mm^2^. Based on the fact that the degree of independent observer concordance was high (Spearman’s rho 0.976, p < 0.001 for cytoplasmic NR4A1, Supplementary Figure 7a and Spearman’s rho 0.960, p < 0.001 for cleaved caspase 3, Supplementary Figure 7b), the median was used for all further analyses.

### Isolation of germinal centre B-cells as non-neoplastic controls

Tonsils from young patients undergoing routine tonsillectomy were disaggregated and separated by Ficoll (GE Healthcare, USA). The specific monoclonal antibodies used were anti CD20 APC (clone: 2H7, order number: 559776) and anti CD77 FITC (clone: 5B5 order number: 551353) all from BD Biosciences. Cells were sorted using the FACSAria (BD Biosciences, Germany) into germinal centre B-cells (GC-B, CD20+, CD77+).

### RNA extraction and RQ-PCR

Total RNA was extracted using Trizol (Invitrogen, Carlsbad, CA, USA) according to the manufacturer’s protocol. cDNA was synthesized using 1 µg total RNA and the RevertAid™ H Minus First Strand cDNA Synthesis Kit (Fermentas, Darmstadt, Germany). Real-time semi quantitative PCR (RQ-PCR) for XPO1, CDKN1B, CDKN1A, GADD45, BCL6, CCNG2, CCNB1, CAT, SOD2, PLK1, EGR3, cFOS, BUB1, MXD1, JUNB, cJUN, ETV5, DUSP1, CCL22, CCR7, CD44, IL10, MMP2, FN1, COL1A, CFLAR, ADARB, EIF4E and EIF4EBP1 (Qiagen, Hilden, Germany, assays and primers are listed in Supplementary Table 6) was performed using an ABI Prism 7000 Detection system (Applied Biosystems, Foster City, CA, USA). PCR reactions were performed in triplicates of 20 µl each. Reaction mix contained 1 × QuantiNova® SYBR Green PCR Kit (Qiagen, Hilden, Germany), 1 mM forward and reverse primer (Eurofins Genomic, Ebersberg, Germany) or 1x QuantiTect Primer Assay (Qiagen, Hilden, Germany), 4 μl cDNA (diluted 1:20) and *Aqua bidest*. up to 20 µl, respectively. GAPDH, PPIA and HPRT, possessing a high correlation coefficient (Spearman rho >0.85 and p < 0.05), served as housekeeping genes. Results are expressed as relative units based on calculation 2^−ΔΔCT^ giving the relative amount of target gene normalized to the endogenous control (geometric mean of the three housekeeping genes) and relative to a low cytoplasmic NR4A1 expressing DLBCL specimen. The cycling protocol included 34 cycles with 2 minutes activation at 95 °C, followed by a denaturation step for 5 seconds at 95 °C and an annealing/extension step for 10 seconds at 60 °C. Melt curve analysis served to identify the different reaction products including nonspecific ones.

### Microarray Analysis

The dataset E-GEOD-10846 (Affymetrix GeneChip Human Genome U133 Plus 2.0)^3^ was downloaded from ArrayExpress and analysed in R 3.3.3^56^. By applying rma, the data was preprocessed with the R package ‘affy’^60,61^, and the 200 clinical samples from R-CHOP-treated patients diagnosed with NGCB (n = 93) or GCB DLBCL (n = 107) were used for the analysis. The R package ‘limma’^62^ was applied to compute differentially expressed genes between the NGCB and GCB subtypes (classification based on the expression profiles of the microarray analysis performed by Lenz *et al*.^3^), and the resulting p-values were adjusted for multiple testing with Benjamini and Hochberg’s method to control the false discovery rate^63^.

Genes with an adjusted p-value < = 0.05 and abs (log_2_FC) >log_2_(1.5) were used as input for the Gene Ontology (GO) analysis (959 unique genes with Entrez IDs, up- and downregulated genes were analysed together). The R package ‘GOstats’ v.2.40.0^64^ was applied to compute hypergeometric p-values for overrepresentation of each GO term (biological process). Terms with a p-value < = 0.01 were considered as significantly enriched (due to the highly dependent nature of the GO, the unadjusted p-values were used but a lower cut-off of 0.01 was applied). Terms were filtered based on the distance to the root ( > = 14) to obtain informative terms. Pathway analysis based on a gene set enrichment analysis (GSEA) was conducted using the R package ‘ReactomePA’ v.1.18.1^65^ and Reactome pathways (v.1.58.0) with a q-value < = 0.05 were considered as significantly enriched. Based on the significantly enriched pathways a gene/pathway matrix was generated. Hierarchical clustering with Euclidean distance and complete linkage was performed and visualized as a heatmap. For the clustering only thoses genes were used, which were contained in the core enrichment as determined by the GSEA and were associated with at least 5 pathways.

### Statistical analysis

Statistical analysis was performed using the IBM SPSS Statistics 23.0 (IBM, Vienna, Austria). P-values < 0.05 were considered statistically significant. The non-parametric Mann–Whitney U-test was used to analyse differences in the expression analysis. Fisher’s exact test was used to determine whether the frequency of cytoplasmic NR4A1 was associated with any clinical parameter. Furthermore, Spearman correlation was applied to estimate the correlation of Caspase 3 and cytoplasmic NR4A1.

## Electronic supplementary material

Supplementary Figure S1–7, Supplementary Table S1 S6

Supplemenetary table 2–5

## References

[1] Pasqualucci L, Dalla-Favera R (2015). The genetic landscape of diffuse large B-cell lymphoma. Seminars in hematology.

[2] Bouska A (2014). Genome-wide copy-number analyses reveal genomic abnormalities involved in transformation of follicular lymphoma. Blood.

[3] Lenz G, Staudt LM (2010). Aggressive lymphomas. The New England journal of medicine.

[4] de Jong D, Balague Ponz O (2011). The molecular background of aggressive B cell lymphomas as a basis for targeted therapy. The Journal of pathology.

[5] Chang C, Kokontis J, Liao SS, Chang Y (1989). Isolation and characterization of human TR3 receptor: a member of steroid receptor superfamily. Journal of steroid biochemistry.

[6] Deutsch AJ, Angerer H, Fuchs TE, Neumeister P (2012). The nuclear orphan receptors NR4A as therapeutic target in cancer therapy. Anticancer Agents Med Chem.

[7] Wenzl K, Troppan K, Neumeister P, Deutsch AJ (2015). The nuclear orphan receptor NR4A1 and NR4A3 as tumor suppressors in hematologic neoplasms. Current drug targets.

[8] Mullican SE (2007). Abrogation of nuclear receptors Nr4a3 and Nr4a1 leads to development of acute myeloid leukemia. Nature medicine.

[9] Deutsch AJ (2014). NR4A1-mediated apoptosis suppresses lymphomagenesis and is associated with a favorable cancer-specific survival in patients with aggressive B-cell lymphomas. Blood.

[10] Lenz G (2008). Stromal gene signatures in large-B-cell lymphomas. The New England journal of medicine.

[11] Deutsch AJA (2017). NR4A3 Suppresses Lymphomagenesis through Induction of Proapoptotic Genes. Cancer research.

[12] Li H (2000). Cytochrome c release and apoptosis induced by mitochondrial targeting of nuclear orphan receptor TR3. Science.

[13] Lin B (2004). Conversion of Bcl-2 from protector to killer by interaction with nuclear orphan receptor Nur77/TR3. Cell.

[14] Thompson J, Burger ML, Whang H, Winoto A (2010). Protein kinase C regulates mitochondrial targeting of Nur77 and its family member Nor-1 in thymocytes undergoing apoptosis. European journal of immunology.

[15] Alizadeh AA (2000). Distinct types of diffuse large B-cell lymphoma identified by gene expression profiling. Nature.

[16] Rosenwald A (2002). The use of molecular profiling to predict survival after chemotherapy for diffuse large-B-cell lymphoma. The New England journal of medicine.

[17] Grill C (2004). Analysis of the ERK1,2 transcriptome in mammary epithelial cells. Biochem J.

[18] Manning BD, Cantley LC (2007). AKT/PKB signaling: navigating downstream. Cell.

[19] Kumar, N. *et al*. Prostate Cancer Chemoprevention Targeting High Risk Populations: Model for Trial Design and Outcome Measures. *Journal of cancer science & therapy* 2011 (2012). 10.4172/1948-5956.s3-007 PMC3300067 22422102

[20] Birkenkamp KU, Coffer PJ (2003). Regulation of cell survival and proliferation by the FOXO (Forkhead box, class O) subfamily of Forkhead transcription factors. Biochemical Society transactions.

[21] Schmid CA (2015). DUSP4 deficiency caused by promoter hypermethylation drives JNK signaling and tumor cell survival in diffuse large B cell lymphoma. The Journal of experimental medicine.

[22] Gururajan M (2005). c-Jun N-terminal kinase (JNK) is required for survival and proliferation of B-lymphoma cells. Blood.

[23] Ezell SA (2016). Differential regulation of mTOR signaling determines sensitivity to AKT inhibition in diffuse large B cell lymphoma. Oncotarget.

[24] Xu ZZ (2013). Activation of the PI3K/AKT/mTOR pathway in diffuse large B cell lymphoma: clinical significance and inhibitory effect of rituximab. Annals of hematology.

[25] Majchrzak A, Witkowska M, Smolewski P (2014). Inhibition of the PI3K/Akt/mTOR signaling pathway in diffuse large B-cell lymphoma: current knowledge and clinical significance. Molecules.

[26] Alain T (2012). eIF4E/4E-BP ratio predicts the efficacy of mTOR targeted therapies. Cancer research.

[27] Kolluri SK (2003). Mitogenic effect of orphan receptor TR3 and its regulation by MEKK1 in lung cancer cells. Molecular and cellular biology.

[28] Slagsvold HH, Ostvold AC, Fallgren AB, Paulsen RE (2002). Nuclear receptor and apoptosis initiator NGFI-B is a substrate for kinase ERK2. Biochem Biophys Res Commun.

[29] Wang A (2009). Phosphorylation of Nur77 by the MEK-ERK-RSK cascade induces mitochondrial translocation and apoptosis in T cells. Journal of immunology.

[30] Mittelstadt PR, DeFranco AL (1993). Induction of early response genes by cross-linking membrane Ig on B lymphocytes. Journal of immunology.

[31] Zikherman J, Parameswaran R, Weiss A (2012). Endogenous antigen tunes the responsiveness of naive B cells but not T cells. Nature.

[32] Inamoto T, Czerniak BA, Dinney CP, Kamat AM (2010). Cytoplasmic mislocalization of the orphan nuclear receptor Nurr1 is a prognostic factor in bladder cancer. Cancer.

[33] Lee JM, Lee KH, Weidner M, Osborne BA, Hayward SD (2002). Epstein-Barr virus EBNA2 blocks Nur77- mediated apoptosis. Proceedings of the National Academy of Sciences of the United States of America.

[34] Mapara MY (1995). Involvement of NAK-1, the human nur77 homologue, in surface IgM-mediated apoptosis in Burkitt lymphoma cell line BL41. European journal of immunology.

[35] Villeneuve DJ (2006). cDNA microarray analysis of isogenic paclitaxel- and doxorubicin-resistant breast tumor cell lines reveals distinct drug-specific genetic signatures of resistance. Breast Cancer Res Treat.

[36] Cittera E (2005). Rituximab induces different but overlapping sets of genes in human B-lymphoma cell lines. Cancer immunology, immunotherapy: CII.

[37] Franke A, Niederfellner GJ, Klein C, Burtscher H (2011). Antibodies against CD20 or B-cell receptor induce similar transcription patterns in human lymphoma cell lines. PloS one.

[38] Arai M, Sasaki A, Saito N, Nakazato Y (2005). Immunohistochemical analysis of cleaved caspase-3 detects high level of apoptosis frequently in diffuse large B-cell lymphomas of the central nervous system. Pathology international.

[39] Muris JJ (2005). Immunohistochemical profiling of caspase signaling pathways predicts clinical response to chemotherapy in primary nodal diffuse large B-cell lymphomas. Blood.

[40] Jardin F (2016). Recurrent mutations of the exportin 1 gene (XPO1) and their impact on selective inhibitor of nuclear export compounds sensitivity in primary mediastinal B-cell lymphoma. American journal of hematology.

[41] Yoshimura M (2014). Induction of p53-mediated transcription and apoptosis by exportin-1 (XPO1) inhibition in mantle cell lymphoma. Cancer science.

[42] Falini B (2005). Cytoplasmic nucleophosmin in acute myelogenous leukemia with a normal karyotype. The New England journal of medicine.

[43] Puente XS (2011). Whole-genome sequencing identifies recurrent mutations in chronic lymphocytic leukaemia. Nature.

[44] Lenz G (2008). Molecular subtypes of diffuse large B-cell lymphoma arise by distinct genetic pathways. Proceedings of the National Academy of Sciences of the United States of America.

[45] Etchin J (2013). Antileukemic activity of nuclear export inhibitors that spare normal hematopoietic cells. Leukemia.

[46] Lapalombella R (2012). Selective inhibitors of nuclear export show that CRM1/XPO1 is a target in chronic lymphocytic leukemia. Blood.

[47] Das A, Wei G, Parikh K, Liu D (2015). Selective inhibitors of nuclear export (SINE) in hematological malignancies. Experimental hematology & oncology.

[48] Kuruvilla J (2017). Selective inhibition of nuclear export with selinexor in patients with non-Hodgkin lymphoma. Blood.

[49] Han YH (2006). Regulation of Nur77 nuclear export by c-Jun N-terminal kinase and Akt. Oncogene.

[50] Swanson KD, Taylor LK, Haung L, Burlingame AL, Landreth GE (1999). Transcription factor phosphorylation bypp90(rsk2). Identification of Fos kinase and NGFI-B kinase I as pp90(rsk2). The Journal of biological chemistry.

[51] Wingate AD, Arthur JS (2006). Post-translational control of Nur77. Biochem Soc Trans.

[52] Wingate AD, Campbell DG, Peggie M, Arthur JS (2006). Nur77 is phosphorylated in cells by RSK in response to mitogenic stimulation. Biochem J.

[53] Campo E (2011). The 2008 WHO classification of lymphoid neoplasms and beyond: evolving concepts and practical applications. Blood.

[54] Hans CP (2004). Confirmation of the molecular classification of diffuse large B-cell lymphoma by immunohistochemistry using a tissue microarray. Blood.

[55] Davies AJ (2007). Transformation of follicular lymphoma to diffuse large B-cell lymphoma proceeds by distinct oncogenic mechanisms. British journal of haematology.

[56] R Development Core Team. R: A language and environment for statistical analysis (2016).

[57] Therneau, T. M. & Grambsch, P. M. Modeling survival data: Extending the Cox model. 1st edn, (Springer-Verlag New York, 2000).

[58] Kassambara, A. & Kosinski, M. survminer: Drawing Survival Curves using ‘ggplot2’ (2017).

[59] Gordon, M. & Seifert, R. Regression Helper Functions. (R package version1.2., August 29, 2016).

[60] Gautier L, Cope L, Bolstad BM, Irizarry R (2004). A. affy–analysis of Affymetrix GeneChip data at the probe level. Bioinformatics.

[61] Abell A. N., Granger D. A., Johnson N. L., Vincent-Jordan N., Dibble C. F., Johnson G. L. (2009). Trophoblast Stem Cell Maintenance by Fibroblast Growth Factor 4 Requires MEKK4 Activation of Jun N-Terminal Kinase. Molecular and Cellular Biology.

[62] Ritchie ME (2015). limma powers differential expression analyses for RNA-sequencing and microarray studies. Nucleic acids research.

[63] Benjamini Y, Hochberg Y (1995). Controlling the False Discovery Rate: A Practical and Powerful Approach to Multiple Testing. Journal of the Royal Statistical Society. Series B (Methodological).

[64] Falcon S (2007). & Gentleman, R. Using GOstats to test gene lists for GO term association. Bioinformatics.

[65] Yu G, He QY (2016). ReactomePA: an R/Bioconductor package for reactome pathway analysis and visualization. Mol Biosyst.

