# Supplementary material for: Cytoplasmic location of NR4A1 in aggressive lymphomas is associated with a favourable cancer specific survival
Source: Sci Rep. 2018 Sep 28;8:14528. doi: 10.1038/s41598-018-32972-4 (PMC6162226; doi:10.1038/s41598-018-32972-4)
Supplement: Supplementary file 1 — Supplementary Figure S1–7, Supplementary Table S1 S6 [file 41598_2018_32972_MOESM1_ESM.pdf]

Cytoplasmic location of NR4A1 in aggressive lymphomas is associated with a favourable cancer specific survival

Karoline Fechter, Julia Feichtinger, Katharina Prochazka, Julia Judith Unterluggauer, Katrin Pansy, Elisabeth Steinbauer, Martin Pichler, Johannes Haybaeck, Andreas Prokesch, Hildegard T. Greinix, Christine Beham-Schmid, Peter Neumeister, Gerhard G. Thallinger, and Alexander J. A. Deutsch

Supplementary Figure 1

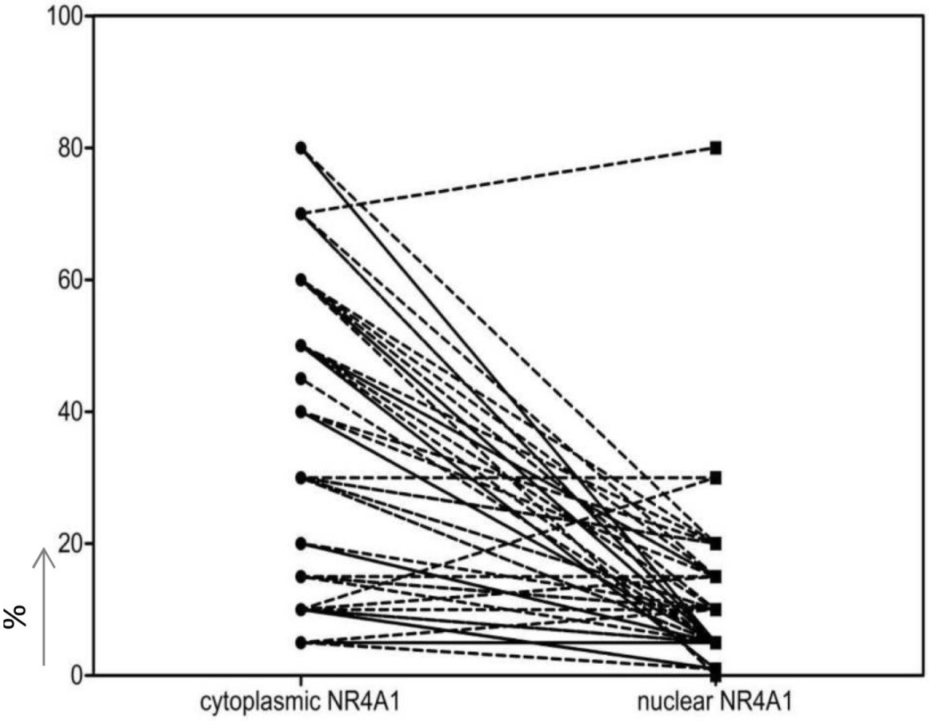

Scatter plot of the correlation of cytoplasmic and nuclear NR4A1 content. Each line represents the relation of cytoplasmic and nuclear NR4A1 protein expression in one respective sample of the DLBCL cohort (n=60).

Supplementary Figure 2

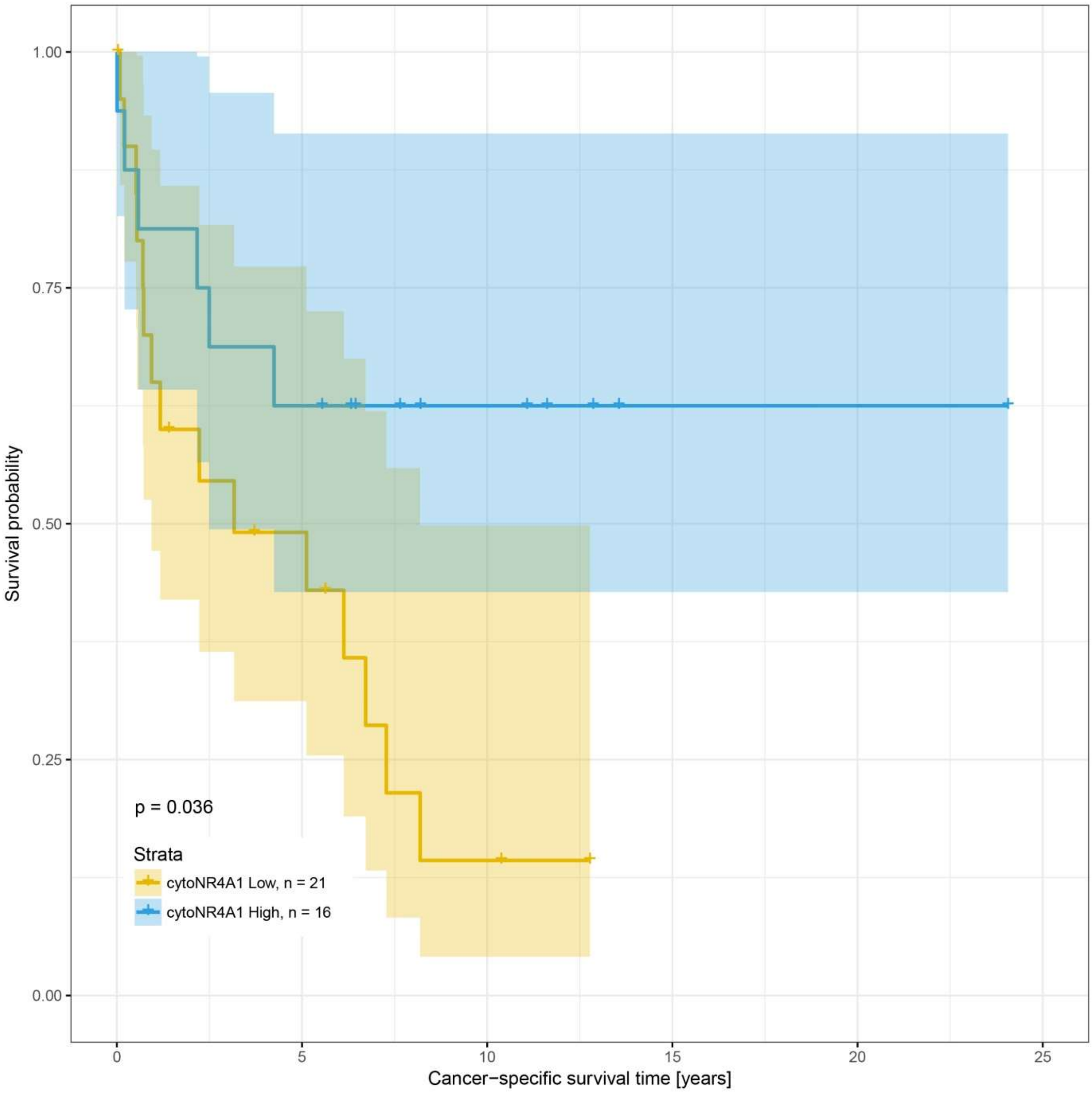

Kaplan-Meier graphical illustration of the cancer-specific survival of de novo DLBCL patients. Patients with high levels of cytoplasmic NR4A1 are depicted in blue and with low levels in yellow

Supplementary Figure 3

a

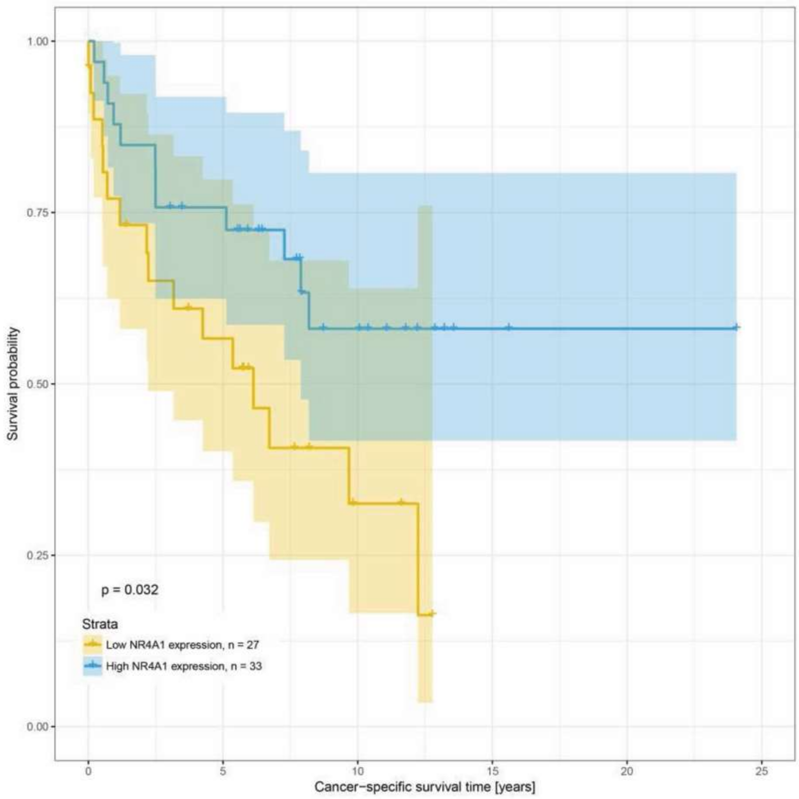

b

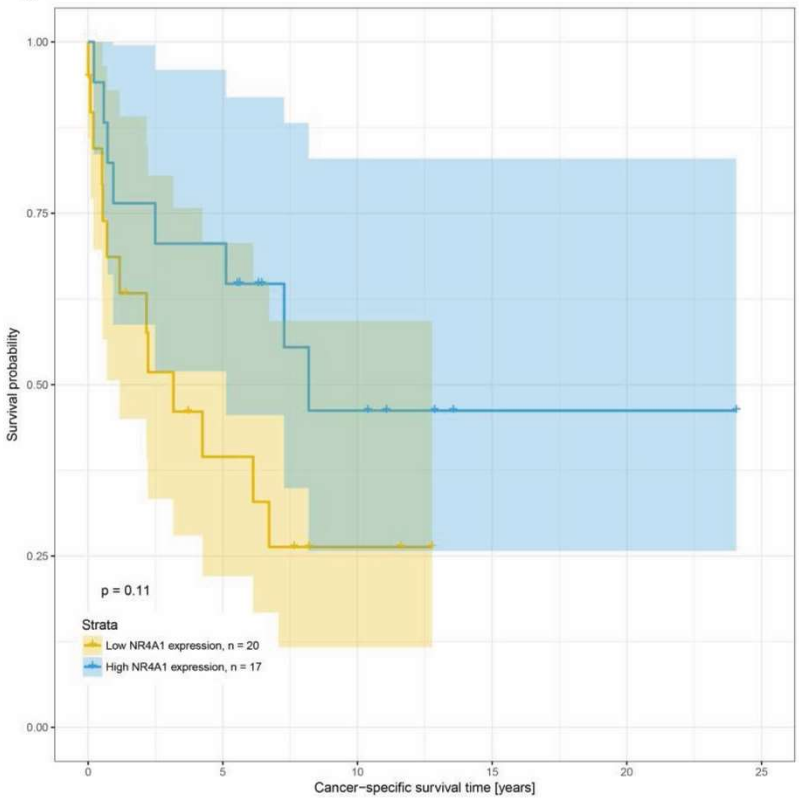

Kaplan-Meier graphical illustration of the cancer-specific survival classified by *NR4A1* expression of lymphoma patients. (a) Kaplan-Meier graphical illustration of all DLBCL patients. High *NR4A1* expression correlates with better survival (p=0.032), which we described in our previous work. (b) Kaplan-Meier graphical illustration of the *de novo* DLBCL patients. We have updated the figure to incorporate the extended follow-up time. Patients with high *NR4A1* expression are shown in blue and with low expression in yellow.

Supplementary Figure 4

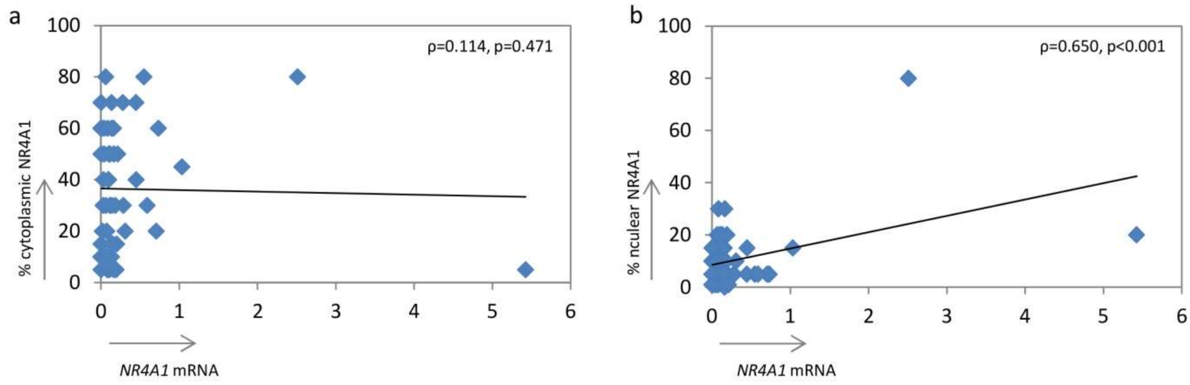

Regression analysis of cytoplasmic and nuclear NR4A1 content in relation to *NR4A1* mRNA expression levels. (a). Relation of cytoplasmic NR4A1 protein abundance to mRNA expression levels (ρ=0.114, p=0.471). (b) Relation of nuclear NR4A1 protein abundance to mRNA expression levels (ρ=0.650, p<0.001).

Supplementary Figure 5

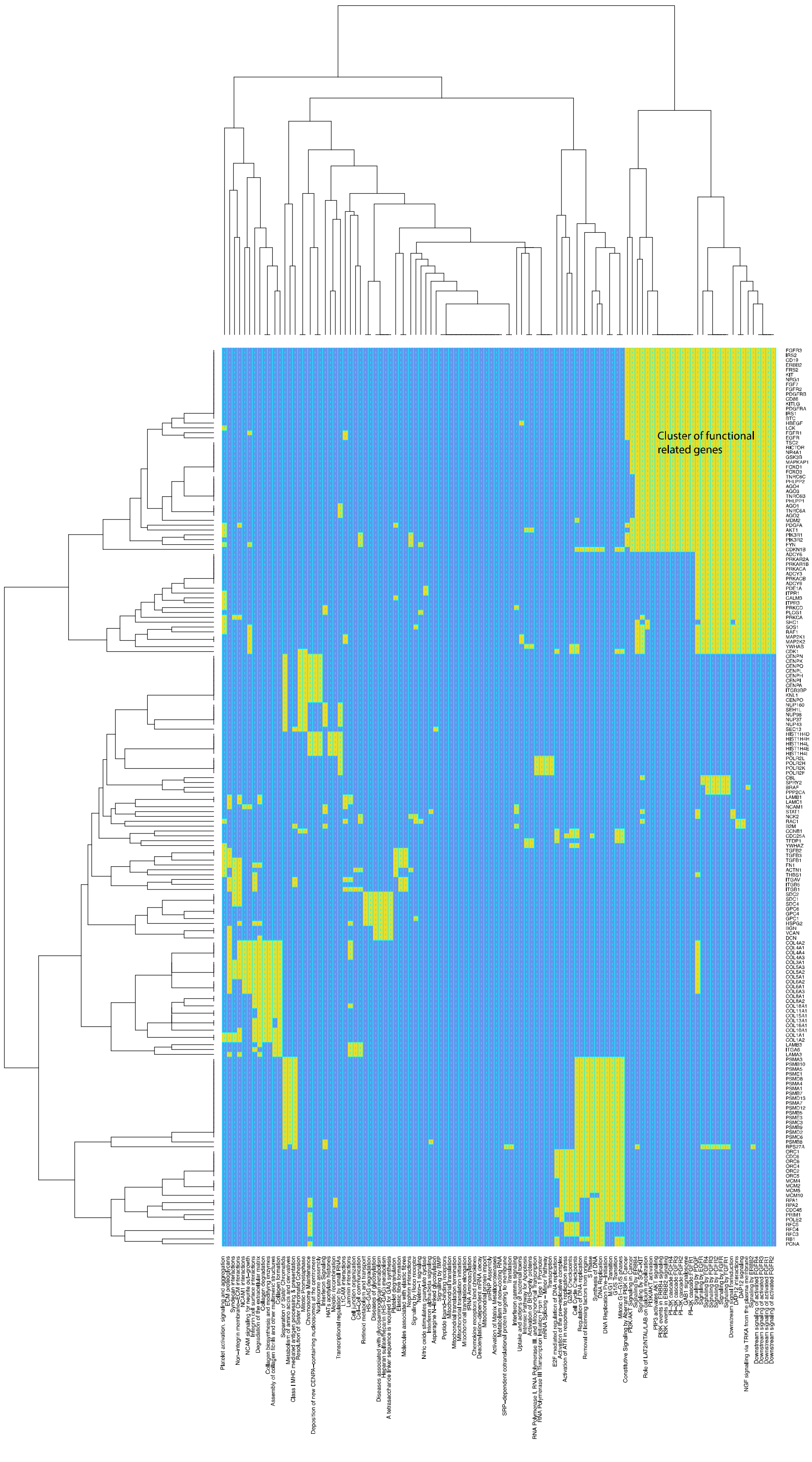

Hierarchical clustering of enriched pathways and associated genes, visualized as a heatmap. A functional related group is denoted in the figure. Genes associated with a given pathway are presented in yellow. Genes not associated with a given pathway are shown in blue.

Supplementary Figure 6

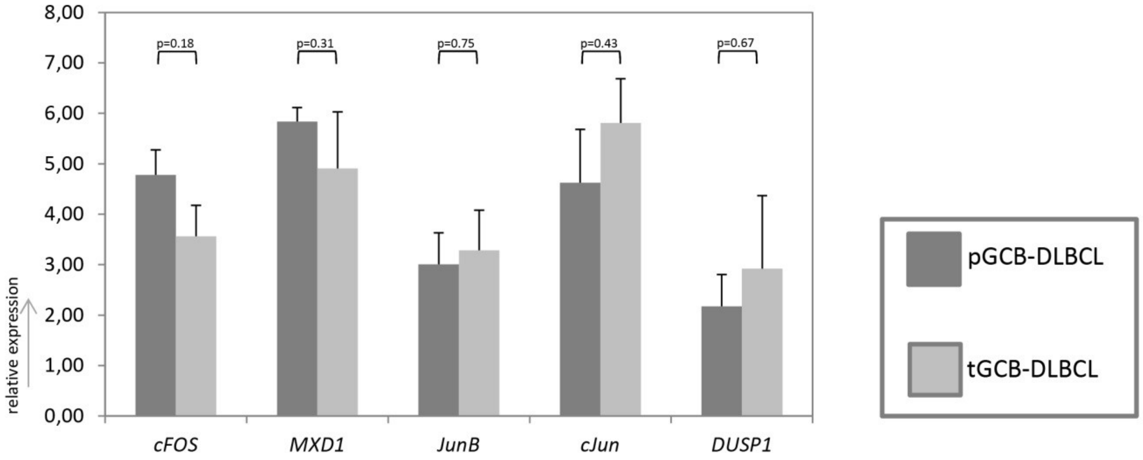

Expression analysis of ERK1/2 target genes. (a) Relative expression levels of *cFOS*, *MXD*, *JUN B*, *cJUN* and *DUSP1* in *de novo* (pGCB-DLBCL, n=9) and transformed (tGCB-DLCL, n=20) GCB-DLBCL determined by RQ-PCR. Each bar represents the mean values of expression levels ± standard error of the mean (SEM).

Supplementary Figure 7

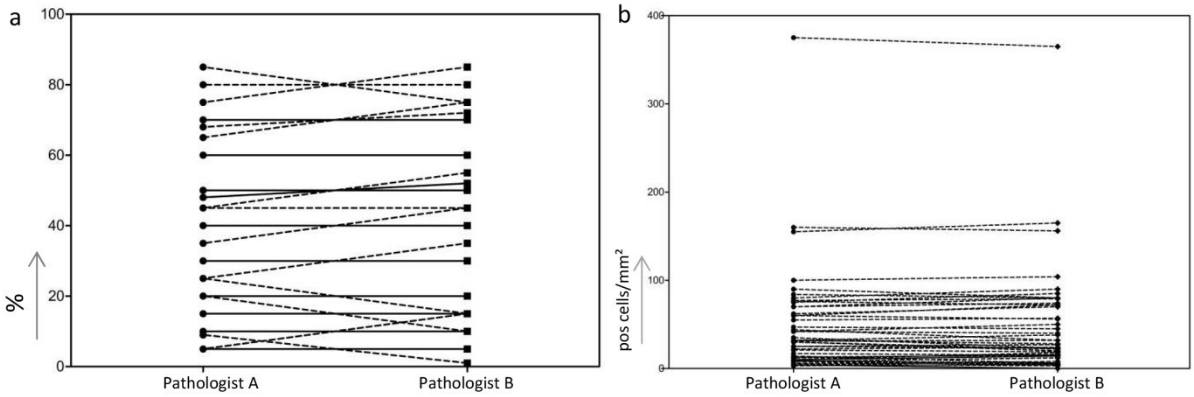

Correlation of independent observers for determination of cytoplasmic NR4A1 and cleaved caspase 3. (a). Accordance between two independent pathologists (Pathologist A and Pathologist B) in determination of the percentage of cytoplasmic NR4A1 content in IHC samples of the DLBCL cohort (n=60, ρ=0.976, p<0.001). (b) Accordance between two independent pathologists (Pathologist A and Pathologist B) in determination of the number of positive cells/ mm^2 in IHC samples of the DLBCL cohort (n=60, ρ=0.960, p<0.001).

Supplementary Table 1: Clinico-pathologic characteristics of the patients included in this study

Clinico-pathologic parameters	Patients (n=60)	Proportion
**Gender**		
Male	31	52%
Female	29	48%
**Age at diagnosis**		
<65	27	45%
Male	15	55.6%
Female	12	44.4%
>65	33	55%
Male	16	48.5%
Female	17	51.5%
**Ann Arbor Stage**		
1	10	16.7%
2	11	18.3%
3	21	35%
4	14	23.3%
Unclassified	4	6.7%
**IPI (International Prognostic Index)**		
1	19	31.7%
2	14	23.3%
3	12	20%
4	11	18.3%
Unclassified	4	6.7%
**Immunophenotype (Hans algorithm)**		
Non-germinal center B-cell like	22	36.67%
Germinal center B-cell like	35	58.33%
pGCB-DLBCL	12	20%
tGCB-DLBCL	23	38.33%
Unclassified	3	5%

Supplementary Table 6: Oligonucleotide sequences of primers for RQ-PCR

Gene - Primer ID	Sequence
GAPDH3428-f<br>GAPDH3428-r	AAG GTC GGA GTC AAC GGA TTT<br>ACC AGA GTT AAA AGC AGC CCT G
HPRT1-f<br>HPRT1-r	ATG GGA GGC CAT CAC ATT<br>ATG TAA TCC AGC AGG TCA GCA A
PPIA-f<br>PPIA-r	CTC CTT TGA GCT GTT TGC AG<br>CAC CAC ATG CTT GCC ATC C
ElF4E-f<br>EIF4E-r	TGC GGC TGA TCT CCA AGT TTG<br>CCC ACA TAG GCT CAA TAC CAT C
ElF4EBP1-f<br>EIF4EBP1-r	CAC CCC GGG AGG TAC CAG GAT C<br>CGC CCG CCC GCT TAT CTT CT
XPO1	Qiagen # QT00045528
CDKN1B	Qiagen #QT00998445
CDKN1A	Qiagen #QT00062090
GADD45	Qiagen #QT00014084
BCL6	Qiagen #QT00079233
CCNG2	Qiagen #QT00998193
CCNB1	Qiagen #QT00006615
CAT	Qiagen #QT00079674
SOD2	Qiagen #QT 01008693
PLK1	Qiagen #QT00049749
EGR3	Qiagen #QT00246498
cFOS	Qiagen #QT00007070
BUB1	Qiagen #QT00082929
MXD1	Qiagen #QT00082915
JUNB	Qiagen #QT00201341
cJUN	Qiagen #QT00242956
ETV5	Qiagen #QT00009485
DUSP1	Qiagen #QT00036638
CCL22	Qiagen #QT00089817
CCR7	Qiagen #QT01666686
CD44	Qiagen #QT00073549
IL10	Qiagen #QT00041685
MMP2	Qiagen #QT00088396
FN1	Qiagen #QT00038024
COL1A	Qiagen #QT00037793
CFLAR	Qiagen #QT00064554
ADARB	Qiagen #QT00081655
